# Cisplatin-induced expression of Gb3 enables verotoxin-1 treatment of cisplatin resistance in malignant pleural mesothelioma cells

**DOI:** 10.1038/sj.bjc.6605467

**Published:** 2009-12-15

**Authors:** D Johansson, C Andersson, J Moharer, A Johansson, P Behnam-Motlagh

**Affiliations:** 1 Department of Medical Biosciences, Clinical Chemistry, Umeå University, Umeå S-901 85, Sweden; 2 Department of Odontology, Periodontology, Umeå University, Umeå S-901 85, Sweden; 3 Department of Radiation Sciences, Oncology, Umeå University, Umeå S-901 85, Sweden

**Keywords:** acquired resistance, apoptosis, cisplatin, Gb3, mesothelioma, verotoxin-1

## Abstract

**Background:**

A major problem with cisplatin treatment is the development of acquired-drug resistance of the tumour cells. Verotoxin-1 (VT-1) exerts its cytotoxicity by targeting the membrane glycolipid globotriasosylceramide (Gb3), a molecule associated with drug resistance. Cisplatin- and VT-1-induced apoptosis involves mitogen-activated protein kinase (MAPK) activation, and deactivation of MAPKs is associated with cisplatin resistance. This study aimed to investigate whether a sub-toxic concentration of VT-1 could enhance cisplatin-induced apoptosis and overcome acquired-cisplatin resistance in cultured cancer cell lines.

**Method:**

P31 and H1299 cells with corresponding cisplatin-resistant sub-lines (P31res/H1299res) were incubated with VT-1 and/or cisplatin followed by determination of Gb3 expression, cell viability, apoptosis, and signalling pathways.

**Results:**

Cells from the resistant sub-lines had elevated Gb3 expression compared with the parental cell lines, and cisplatin further increased Gb3 expression, whereas VT-1 reduced the percentage of Gb3-expressing cells. Combination of cisplatin and sub-toxic concentrations of VT-1 led to a super-additive increase of cytotoxicity and TUNEL staining, especially in the cisplatin-resistant sub-lines. Blockade of Gb3 synthesis by a Gb3 synthesis inhibitor not only led to eradicated TUNEL staining of P31 cells, but also sensitised P31res cells to the induction of apoptosis by cisplatin alone. Cisplatin- and VT-1-induced apoptosis involved the MAPK pathways with increased C-Jun N-terminal kinase and MAPK kinase-3 and -6 phosphorylation.

**Conclusions:**

We show the presence of Gb3 in acquired-cisplatin resistance in P31res and H1299res cells. Cisplatin up-regulated Gb3 expression in all cells and thus sensitised the cells to VT-1-induced cytotoxicity. A strong super-additive effect of combined cisplatin and a sub-toxic concentration of VT-1 in cisplatin-resistant malignant pleural mesothelioma cells were observed, indicating a new potential clinical-treatment approach.

Lung cancer is the first and second leading cause of cancer-related death in men and women, respectively (Parkin, 2001; Jemal *et al*, 2003). The most common type is non-small cell lung cancer (NSCLC), which accounts for over 75% of all cases (Brognard *et al*, 2001). Asbestos can cause a variety of lung diseases including lung cancer and pleural mesothelioma. Malignant pleural mesothelioma (MPM) is a highly mortal malignancy with poor prognosis partially because of treatment resistance (Leard and Broaddus, 2004). Treatment options are surgery, radiotherapy, and chemotherapy often including platinum-based drugs such as cisplatin (*cis*-diamminedichloroplatinum (II),), which is an extensively used anticancer drug. Cisplatin acts at least in part, by formation of platinum-DNA adducts, which hinders rapidly dividing cells from duplicating their DNA for mitosis and activation of apoptosis (Zwelling *et al*, 1979; Siddik, 2003). However, a major problem with cisplatin treatment is the development of acquired-drug resistance of the cancer cells (Andrews and Howell, 1990; Kasibhatla and Tseng, 2003) involving increased MDR1/PgP activity (Zhou, 2008). Some mechanisms of cisplatin resistance include reduction of platinum accumulation by alteration of transmembrane pumps, enhancement of DNA damage repair, and reduced apoptosis induction (Krishan *et al*, 1997; Cvijic *et al*, 1998; Ohmichi *et al*, 2005; Liu *et al*, 2007). Mitogen-activated protein kinases (MAPKs) are activated in cisplatin-induced apoptosis in most investigated cell systems and induced cisplatin resistance is also associated with reduced activation of MAPKs (Brozovic and Osmak, 2007).

Globotriasosylceramide (Gb3) consist of a trisaccharide linked to a lipid based in the plasma membrane and is expressed by several tumour cell lines originating from breast cancer, ovarian cancer, colon carcinoma, haematological malignancies, and astrocytoma tumours as well as normal endothelial and epithelial cells (LaCasse *et al*, 1999; Gariepy, 2001; Kovbasnjuk *et al*, 2005; Johansson *et al*, 2006). MDR1/PgP acts as a glycolipid translocase involved in the biosynthesis of glycolipids such as Gb3, and elevated levels of Gb3 have also been seen in drug-resistant cancers, and functional interplay between membrane Gb3 and MDR1/PgP has been suggested (Lingwood *et al*, 1998; De Rosa *et al*, 2008). Gb3 functions as cell surface receptor for verotoxin-1 (VT-1, Shiga-like toxin-1) produced by pathogenic strains of *Escherichia coli* (Lingwood *et al*, 1987; Jacewicz *et al*, 1989; Rose and Clark, 1989).

VT-1 has one enzymatically active part (A), and one part that binds to the cell surface (B). The B part consists of five identical sub-units, which can all bind to the Gb3 receptor in which the A sub-unit is internalised and cytotoxic through ribosome inactivation (Endo *et al*, 1988; Olsnes and Sandvig, 1988; Saxena *et al*, 1989; O'Brien *et al*, 1992; Gariepy, 2001; Sandvig *et al*, 2002). VT-1 has shown efficacy against meningioma, astrocytoma, as well as renal tumour xenografts in mice (Arab *et al*, 1999; Salhia *et al*, 2002; Ishitoya *et al*, 2004). The B part of VT-1 has also been suggested as a novel approach to deliver other anti-tumour agents (Vingert *et al*, 2006).

A sub-toxic concentration of VT-1 could possibly enhance cisplatin-induced apoptosis and overcome acquired-cisplatin resistance in cultured cancer cell lines, as MAPKs are involved in apoptosis induction of both agents and in cisplatin resistance. The aim of the study was to quantify Gb3 expression in cisplatin-sensitive and -resistant MPM and NSCLC cell lines, and to investigate the potential of using VT-1 or agent adhered to its B sub-unit as highly potent and specific agents to overcome acquired-cisplatin resistance.

## Materials and methods

### Cell lines and cell culture

Two human cancer cell lines were used: P31 (Marklund *et al*, 1982), an MPM and H1299 (American Type Culture Collection, CRL-5803), an NSCLC cell line as well as corresponding sub-lines with acquired-cisplatin resistance (P31res and H1299res). The cells were maintained under standard cell culture conditions, grown as monolayer culture in Eagle's MEM in Earl's salt (Gibco Ltd, Paisley, Scotland, UK) supplemented by 10% foetal bovine serum (Biochrom KG, Berlin, Germany) and 200 *μ*mol l^–1^ L-glutamine. They were incubated at 37°C in a humidified atmosphere containing 5% CO_2_. Medium of the resistant sub-lines was between experiments supplemented by either 1.2 mg l^–1^ (P31res) or 2 mg l^–1^ (H1299res) cisplatin.

### Determination and inhibition of Gb3 and MDR1/PgP expression of cultured cells

The cellular expression of Gb3 of the cell lines was identified by a monoclonal rat IgM antibody (Immunotech, Marseille, France) and MDR1/PgP by a monoclonal antibody from Chemicon Internat. Inc. (Temecula, CA, USA) on an FACS Calibur flow cytometer (Becton Dickinson Immunocytometry Systems, San Jose, CA). DL-*threo*-1-phenyl-2-palmitoylamino-3-morpholino-1-propanol, PPMP (Sigma-Aldrich, St Louis, MO, USA), a chemical inhibitor of glucosylceramide synthesis, was used to deplete Gb3 expression by culturing cells with 2 *μ*mol l^–1^ PPMP for 72 h. Verapamil was from Abbott Laboratories, Abbott Part, IL USA, and cyclosporin A from Sigma-Aldrich.

### Cell viability assay

A fluorometric method using fluorescein diacetate (Amersham International, Amersham, UK) was used to quantify cell viability and determine VT-1 (Sigma-Aldrich) sensitivity of P31 and H1299 cells *in vitro*. Cells (1 × 10^4^) were plated with 100 mg l^–1^ medium in the wells of 96-well microtiter plates. The plates were first incubated at 37°C for 24 h with culture medium only, then medium was replaced with fresh medium, containing (1) 0.1–5.0 *μ*g l^–1^ (P31) or 0.001–1.0 *μ*g l^–1^ (H1299) VT-1 or (2) 0.1–10.0 mg l^–1^ cisplatin with or without VT-1 (0.1 *μ*g l^–1^ for P31, 0.001 *μ*g l^–1^ for H1299). The incubation was continued for 72 h, and then the medium was removed by flicking the plate, and wells were washed once with 200 *μ*l PBS buffer. To each well was then added 150 *μ*l of PBS containing 10 mg l^–1^ fluorescein diacetate, and the plates incubated for 45 min at 37°C, followed by fluorescence determination in a fluorometer (LS 55, Perkin Elmer, MA, USA) using 485 and 538 nm for excitation and emission, respectively.

### Flow cytometry analysis

P31res and H1299res cells were trypsinised and suspended in PBS and double stained with Gb3 goat anti-rat IgM (Immunotech) and MDR1/PgP IgG_2a_ anti-mouse primary antibodies (Chemicon Millipore, MA USA). Controls were derived by incubating the cells with corresponding rat IgM and mouse IgG_2a_ isotype antibodies (Invitrogen, Carlsbad, CA, USA) for 1 h at 4°C, followed by washing and centrifuging for 10 min with PBS–BSA. Subsequently, cell pellets were re-suspended and incubated with secondary antibody goat anti-rat IgM and goat anti-mouse IgG_2a_ (Invitrogen) for 1 h at 4°C. After washing the cells and centrifuging for 10 min, cells were analysed with an FACScan flow cytometer (Becton Dickinson, San Jose, CA, USA) on channels FL4 or FL1 and data processed using BD Cell Quest software. After gating out debris and cell clumps, the data were plotted as area histograms.

Terminal deoxynucleotidyl transferase (TdT)-mediated dUTP nick end labelling (TUNEL) staining detecting apoptosis-specific nuclear DNA fragmentation was used as a marker for late stage apoptosis. Free 3′-OH terminal was labelled with modified fluorescence-labelled nucleotides (dUTP) by catalysis of TdT. Roche's *in situ* cell death detection kit, TMR red (Roche, Mannheim, Germany), was used. P31 and H1299 cells were cultured to about 80% confluence and the medium was thereafter changed to fresh medium containing 0 or 5 mg l^–1^ cisplatin and/or 0.1 *μ*g l^–1^ VT-1, and incubation continued for 72 h. Cells were thereafter harvested with trypsin and any floating cells were collected by centrifugation. Cells were then TUNEL stained according to the manufactures instructions and TUNEL staining was determined by flow cytometry.

### Caspase activity determination

Fluorometric activity assays measuring caspase-3, -8, and -9 enzyme activities were used (R&D Systems Inc. MN, USA). P31 and H1299 cells were treated with 0 or 5 mg l^–1^ cisplatin and/or 0.1 *μ*g l^–1^ VT-1 for 24 h and thereafter lysed in lysis buffer for 10 min. Cell lysates (total protein concentration 100–200 *μ*g) were incubated with caspase-3, -8, or -9 fluorogenic caspase-specific substrate at 37°C for 2 h. The fluorescence signal was determined with an LS55, Luminescence spectrometer (Perkin Elmer) using 400 and 505 nm excitation and emission wavelengths, respectively. Total protein content was determined with bicinchoninic acid Protein Assay kit (Pierce Biotechnology Inc., IL, USA).

### SDS–PAGE gel electrophoresis and immunoblotting

VT-1 influence on specific proteins involved in apoptosis signal transduction was investigated through western blotting. Cells were exposed to 0 or 5 mg l^–1^ cisplatin and/or 0.1 *μ*g l^–1^ VT-1 for 24 h and then lysed in lysis buffer (R&D Systems Inc.). Cell extract was incubated with NuPAGE 4 × LDS sample buffer and NuPAGE reducing agent for 10 min at 100°C. Samples (19.5 *μ*g l^–1^ protein) were run on a 12.5% Tris–HCl SDS–PAGE criterion precast gel (Bio-Rad, Hercules, CA, USA) using 1 × MOPS buffer and NuPage antioxidant. The buffers, reducing agent, and antioxidant were from Invitrogen. Blotting was performed onto Immune-Blot PVDF membranes (Bio-Rad). Membrane was then blocked in TBS buffer containing 0.2% Tween 20, 20 mmol l^–1^ Tris pH 7.4, and 150 mmol l^–1^ NaCl milk RT for 1 h. Thereafter, the membrane was incubated overnight with primary antibody against Akt, p-Akt (ser473 and tyr308), Bad, p-Bad (ser 136 and 112) Bid, Bim, C-Jun N-terminal kinase (JNK) (1 and 2), p-JNK (1 and 2), MCL-1, MAPK kinase-3 (MKK-3), p-MAPK kinase-3 and -6 (MKK3/6), P44/42, p-P44/42, or PUMA and after repeated washing with TBS buffer, the secondary antibodies both diluted in 5% milk in TBS buffer and 0.25% Tween 20 were incubated for 1 h. Membrane was then washed again with TBS buffer, and antibody detection was performed by enhanced chemiluminescence staining (ECL Advance western blotting detection system, Amersham Biosciences, Buckinghamshire, UK). Monoclonal *β*-actin antibody was used for detection of actin as loading control. All antibodies were from Cell Signalling Technology Inc. (Danvers, MA, USA).

### Statistics

Statistical significance was tested with one-way ANOVA. The level of significance for rejecting the null hypothesis of zero-treatment effect was taken to be *P*=0.05.

## Results

### Basal expression of Gb3 and the effect of cisplatin on MPM and NSCLC cells

**Figure 1:**
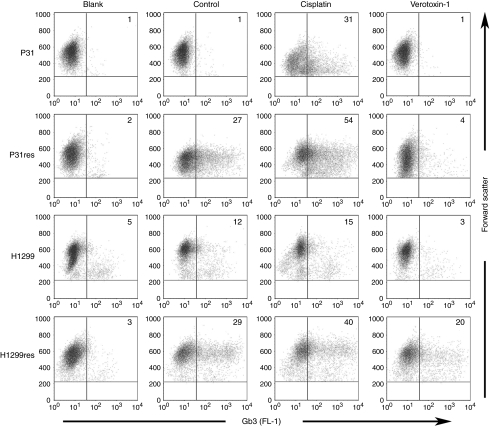
Flow cytometry analysis of Gb3 expression in P31 and H1299 cells. Gb3 expression in cells not incubated with and cells incubated for 72 h with 5 mg l^–1^ cisplatin or 0.1 *μ*g l^–1^ VT-1, respectively. The percentage of Gb3-expressing cells is noted in the right quadrant in each dot plot. Blank shows unspecific secondary anti-body binding, whereas control shows cell not incubated with either cisplatin or VT-1. Representative results out of at least three independent experiments are shown.

Cell surface expression of Gb3 was evaluated by FACS analysis using monoclonal anti-Gb3 antibodies. Low levels of Gb3-expressing cells was found in P31 (1%) and H1299 (12%) cells, but expression was elevated in the cisplatin-resistant sub-lines to 27% and 29%, respectively (Figure 1). Incubation of cells with 5 mg l^–1^ cisplatin for 72 h increased Gb3 expression to 31% and 54% in P31 and P31res cells, and to 15% and 40% in H1299 and H1299res, respectively. Incubation with 0.1 *μ*g l^–1^ VT-1 reduced the percentage of Gb3-expressing P31 and P31res cells to 1% and 4%, respectively, and incubation with 0.001 *μ*g l^–1^ VT-1 markedly reduced the percentage of Gb3-expressing H1299 and H1299res cells to 3% and 20%, respectively (Figure 1).

### VT-1 and cisplatin cytotoxicity on MPM and NSCLC cells

**Figure 2:**
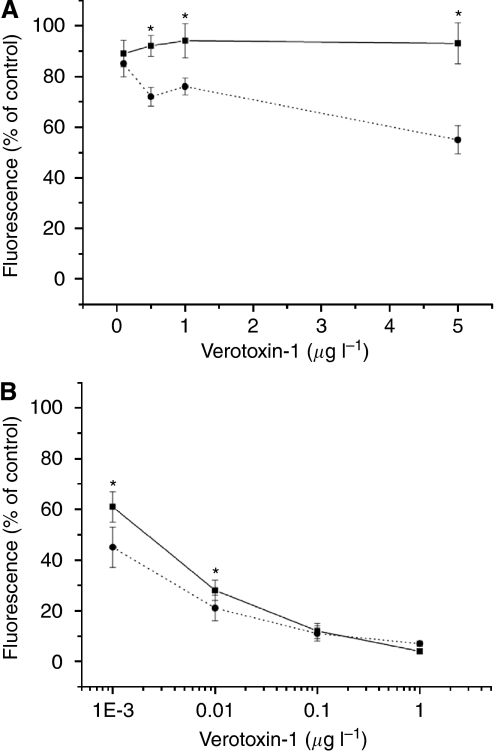
Cell viability (FMCA assay) after exposure to increasing concentrations of VT-1 for 72 h. (**A**) P31 (straight line) and P31res (dotted line) cells exposed to 0.1–5.0 *μ*g l^–1^ VT-1. (**B**) H1299 (straight line) and H1299res (dotted line) cells exposed to 0.001–1 *μ*g l^–1^ VT-1. Significant differences (*P*<0.05) between cell lines is indicated (^*^). Mean±s.d. (*n* =3).

**Figure 3:**
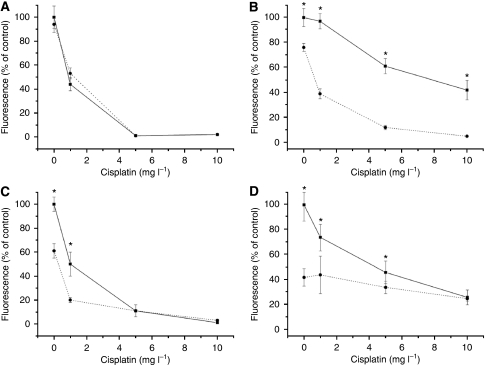
Cell viability (FMCA assay) after exposure of MPM and NSCLC cells to 0.1–10 mg l^–1^ cisplatin alone (filled line) or in combination with 0.1 *μ*g l^–1^ (P31 sub-lines) or 0.001 *μ*g l^–1^ (H1299 sub-lines) of VT-1 (dotted line) for 72 h. (**A**) P31cells, (**B**) P31res cells, (**C**) H1299t cells, and (**D**) H1299res cells. Significant differences (*P*<0.05) between cell lines with cisplatin alone and when combined with VT-1 is indicated (^*^). Mean±s.d. (*n*=3).

Exposure of the MPM cells to 0.1–5 *μ*g l^–1^ VT-1 for 72 h showed no cytotoxicity of the toxin to P31 cells and a modest cytotoxicity on P31res cells, whereas both NSCLC cell sub-types were sensitive to VT-1 in concentrations as low as 1 × 10^−3^ *μ*g l^–1^ (Figure 2). Incubation with 0.1–10 mg l^–1^ cisplatin for 72 h reduced cell viability in P31 and H1299 cells concentration dependently and as expected less so in the cisplatin-resistant sub-lines (Figure 3). Combination of cisplatin and VT-1 (0.1 or 0.001 *μ*g l^–1^) led to a significant increase in cytotoxicity, especially in the cisplatin-resistant sub-lines (Figure 3).

### MDR1/PgP and Gb3 expression of cells and their resistant cell sub-lines

**Figure 4:**
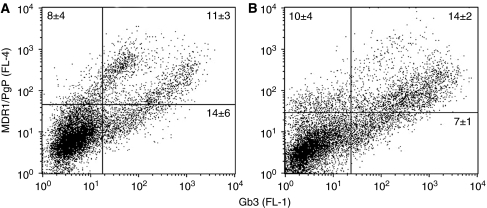
Flow cytometry analysis of Gb3- and MDR1/Pgp expression of cisplatin-resistant cell sub-lines. (**A**) Cell surface expression of MDR1/PgP (ordinate) and Gb3 (abscissa) of P31res cells. (**B**) Cell surface expression of MDR1/PgP (ordinate) and Gb3 (abscissa) of H1299res cells. Representative results are shown and the percentage mean (*n*=3) of stained cells is noted in each dot plot.

Flow cytometry showed a correlation between MDR1/PgP and Gb3 co-expression in P31res as well as H1299res cell sub-lines (Figure 4). P31res cells showed co-expression in two sub-fractions with one expressing ∼10-fold expression of MDR1/PgP compared with Gb3. Incubation of the cells with 10 *μ*mol l^–1^ verapamil for 72 h before expression analysis did, however, not reduce the expression of MDR1/PgP or Gb3 (results not show).

**Figure 5:**
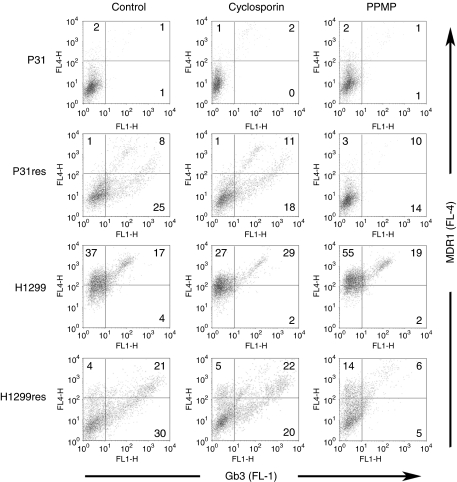
Flow cytometry analysis of Gb3- and MDR1/PgP expression of P31 and H1299 cells and their cisplatin-resistant sub-lines incubated with 10 *μ*mol l^–1^ cyclosporin A or 2 *μ*mol l^–1^ PPMP for 72 h. Representative results are shown and the percentage mean (*n*=3) of stained cells is noted in each dot plot.

We, therefore, also tested whether the more effective MDR1/PgP inhibitor cyclosporin A (10 *μ*mol l^–1^ incubated with the cells for 72 h) as well as PPMP (2 *μ*mol l^–1^) affected the co-expression of MDR1/PgP and Gb3. Un-expectantly, cyclosporin A did not noticeably inhibit MDR1/PgP expression in any of the cell types, but possibly the expression of Gb3 in the resistant sub-lines, whereas PPMP, as expected markedly, reduced not only Gb3 expression in the resistant cell sub-lines, but also of MDR1/PgP expression, especially of the cells of the resistant cell lines with also high expression of Gb3 (Figure 5).

### VT-1 and cisplatin induction of MPM cell DNA fragmentation

**Figure 6:**
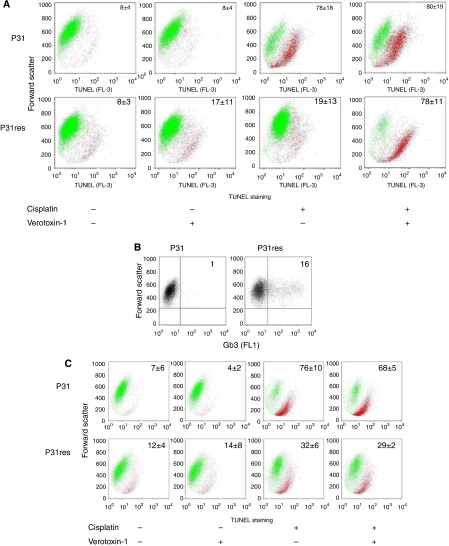
(**A**) Flow cytometry analysis of TUNEL staining in P31 cells after 72 h incubation with 5 mg l^–1^ cisplatin and 0.1 *μ*g l^–1^ VT-1, alone or in combination. Green dots indicate unstained cells and red TUNEL-stained cells. The percentage mean±s.d. (*n*=3) of TUNEL-stained cells is noted in the dot plots. (**B**) Flow cytometry analysis of Gb3 expression in P31 and P31res cells after 72 h incubation with 2 *μ*mol l^–1^ PPMP. The percentage mean±s.d. (*n*=3) of Gb3-stained cells is noted in each dot plot. (**C**) Flow cytometry analysis of TUNEL staining in P31and P31res cells pre-incubated with 2 *μ*mol l^–1^ PPMP, and after 72 h incubation with 5 mg l^–1^ cisplatin and 0.1 *μ*g l^–1^ VT-1, alone or in combination. Green dots indicate unstained cells and red TUNEL-stained cells. The percentage mean±s.d. (*n*=3) of TUNEL-stained cells is noted in each dot plot. Representative results are shown in **A**–**C**.

The TUNEL-staining assay showed no increase of DNA fragmentation in P31 cells after exposure to 0.1 *μ*g l^–1^ VT-1 for 72 h. A slight increase (to 17%) in DNA fragmentation was, however, noted in the P31res cells (Figure 6A). Cisplatin (5 mg l^–1^) was sufficient to induce massive (to 78%) DNA fragmentation in P31 cells, whereas there was no or limited effect (19%) in the resistant sub-line (P31res). The proportion of P31res cells with DNA fragmentation was dramatically increased (to 78% of the cells) by combined exposure to 5 mg l^–1^ cisplatin and 0.1 *μ*g l^–1^ VT-1, but no further effect than that of cisplatin alone was noted in the P31 cells (Figure 6A).

### Inhibition of Gb3 expression eradicates VT-1 super-additive effect on cisplatin-induced TUNEL staining of cisplatin-resistant MPM cells

Exposure to 2 *μ*mol l^–1^ PPMP for 72 h significantly reduced, but did not completely inhibit Gb3 expression in P31res cells (Figure 6B). PPMP treatment for 72 h eradicated DNA fragmentation induced by VT-1 (0.1 *μ*g l^–1^) in combination with cisplatin in P31 cells (Figure 6C). However, PPMP treatment seemed to sensitise P31res cells to the induction of apoptosis by 5 mg l^–1^ cisplatin alone and possibly by itself induced low levels of DNA fragmentation (Figure 6C). Gb3 expression and TUNEL staining of P31 cells were unaffected by PPMP preincubation (Figure 6B and C).

### VT-1 and cisplatin induction of MPM cell caspase activity

**Figure 7:**
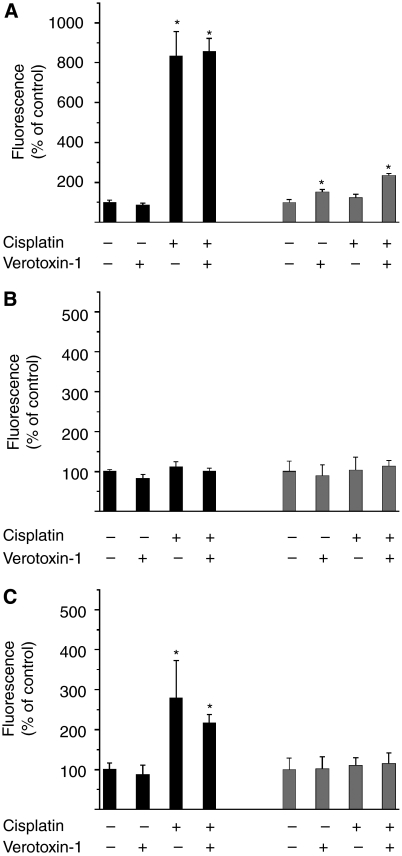
(**A**) Caspase-3, (**B**) -8, and (**C**) -9 enzyme activity in P31 (black bars) and P31res cells (grey bars) after 24 h incubation with 5 mg l^–1^ cisplatin and 0.1 *μ*g l^–1^ VT-1, alone or in combination. Significant enzyme activity differences (*P*<0.05) compared with untreated control is indicated (^*^). Mean±s.d. (*n*=3).

When we studied signal transduction to apoptosis, the enzyme activity assays for caspase-3, -8, and -9 showed cisplatin-induced activation of caspase-3 and -9 in P31cells, but not in P31res cells. VT-1 (0.1 *μ*g l^–1^) activated caspase-3 in P31res, but had no effect on P31cells (Figure 7). When cisplatin was combined with VT-1, no further activation of caspase activity was noted except for slight increase of caspase-3 activity in P31res cells.

### Phosphorylation of MAPK proteins

**Figure 8:**
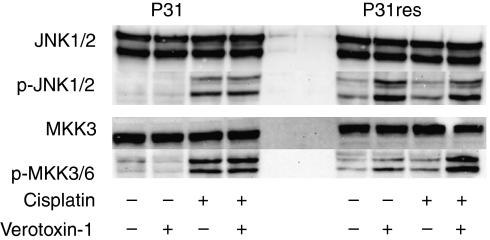
Western blot analysis of total and phosphorylated JNK1/2 and MKK3/6 protein expression of P31and P31res cells after 24 h incubation with 5 mg l^–1^ cisplatin and 0.1 *μ*g l^–1^ VT-1, alone or in combination. Representative blot (*n*=3).

Western blotting was used to study expression of proteins potentially involved in apoptosis signalling of MPM cells after 24 h exposure to 5 mg l^–1^ cisplatin with or without 0.1 *μ*g l^–1^ VT-1. An involvement of the stress-activated MAPK signalling pathway with increased phosphorylation of JNK and MKK3/6 was noted. JNK and MKK3/6 were phosphorylated after cisplatin exposure in P31cells, but not in P31res cells, whereas VT-1 induced phosphorylation in P31res cells, but not in P31cells. MKK3/6 phosphorylation was further augmented by the combination of cisplatin and VT-1 compared with VT-1 alone (Figure 8). Antibodies against AKT, p-Akt (ser473 and tyr308), Bad, p-Bad (ser 136 and 112), Bid, Bim, MCL-1, MKK-3, p-MKK3/6, P44/42, p-P44/42, or PUMA were all tested without conclusive protein expression changes after cisplatin and/or VT-1 exposure (data not shown).

## Discussion

This study shows the presence of the VT-1 cell receptor Gb3 in acquired-cisplatin resistance in both MPM and NSCLC cells. Furthermore, we show that cisplatin sensitises the cells to VT-1, leading to a potential treatment approach. Owing to the frequency of and the rapid acquirement of cisplatin resistance in the clinical setting, effective measures to circumvent this major treatment obstacle are of outmost importance.

We showed that cisplatin can up-regulate Gb3 expression in MPM and NSCLC cells and thus sensitise the cells to VT-1-induced cytotoxicity. The increased proportion of Gb3-expressing cells after cisplatin treatment suggests that cisplatin induces Gb3 expression in cancer cells, that cisplatin preferentially eradicates cell with low Gb3 expression, and that Gb3 expression is linked to acquired-cisplatin resistance. We could also correlate increased expression of Gb3 of cisplatin-resistant MPM and NSCLC cells to increased expression of MDR1/PgP. Gb3 and MDR1/PgP have recently been found to be partially co-localised in MDR1/PgP-expressing cells, and Gb3-containing lipid rafts are important for intracellular MDR1/PgP surface trafficking (De Rosa *et al*, 2008). The increased expression of MDR1/PgP in cisplatin resistance could, therefore, parallel increased Gb3 expression, as MDR1/PgP acts as a glycolipid translocase involved in the biosynthesis of glycolipids such as Gb3 (De Rosa *et al*, 2008). However, we found no effect of the MDR1/PgP inhibitors verapamil or cyclosporin A on the expression of MDR1/PgP in either cell line, but PPMP reduced Gb3 expression in resistant sub-line cells and interestingly also particularly of the Gb3-expressing fraction that was induced when the mother cell line was made cisplatin resistant. Further studies on the interrelationship between multidrug-resistant cell expression of drug efflux pumps and Gb3 on cisplatin-resistant MPM and NSCLC cells are ongoing.

The reduced amount of Gb3-expressing cells after VT-1 treatment confirms that high-Gb3-expressing and cisplatin-resistant cells are sensitive to VT-1. Even though VT-1 alone had limited effect on the whole-cell population, it is of interest to note that there was a strong super-additive effect of combined cisplatin and VT-1 treatment in cisplatin-resistant MPM cells. The high sensitivity of the NSCLC cells to VT-1 despite a modest Gb3 expression implies that Gb3 expression, though necessary, does not mediate VT-1 cytotoxicity alone. The increased expression of Gb3 parallel to MDR1/PgP expression after induced cisplatin resistance with ensuing sensitivity to VT-1 cytotoxicity needs further investigation. It is in this context, however, that intriguing blockade of Gb3 synthesis eradicated VT-1-induced apoptosis as well as re-sensitised P31res cells to the induction of apoptosis by cisplatin alone.

We continued the study by investigating cisplatin- and VT-1-induced cell death signal pathways in the MPM cells. TUNEL-labelled DNA-fragmentation analysis showed an induced increase of apoptosis that correlated well with cell cytotoxicity and confirmed that the cisplatin-resistant sub-line was significantly less sensitive to cisplatin-induced apoptosis. Despite that neither VT-1 nor cisplatin induced more than limited amount of apoptosis in the cisplatin-resistant sub-line, the combined treatment induced similar levels of apoptosis as seen with cisplatin treatment alone of the more cisplatin-sensitive parental cell line. The lack of cisplatin-induced DNA fragmentation in the resistant sub-line correlated with the results from the caspase activity assays, as there was no activation of either caspase-3, -8, or -9 despite significant activation of caspases-3 and -9 in the parental cell line. VT-1 did, however, slightly activate caspase-3 activity in the resistant cells, which was further increased by combined VT-1/cisplatin treatment. This could explain the increased DNA fragmentation, especially as it has been shown that low levels of caspase-3 activity is enough to induce apoptosis in these cells (Johansson *et al*, 2008). PPMP inhibition of Gb3 synthesis confirmed that Gb3 is essential for VT-1-enhanced cisplatin-induced apoptosis.

Western blot was used to elucidate the signal transduction pathways to apoptosis of VT-1 and cisplatin alone and combined. By using antibodies specific for phosphorylated proteins of the MAPK pathway, we found that MKK3/6 and JNK was phosphorylated after cisplatin treatment in the cisplatin-sensitive cells, but not in the corresponding sub-lines with acquired-cisplatin resistance. VT-1 induced phosphorylation of MKK3/6, which was enhanced when VT-1 was combined with cisplatin. MKK3/6 is known to activate P38 (Derijard *et al*, 1995; Han *et al*, 1996), and P38 as well as JNK has been shown to promote apoptosis in response to cellular stress (Kim *et al*, 2006). Treatment of cells with chemical inhibitors or siRNA targeting p38 was recently shown to specifically inhibit VT-1 transport to the Golgi and reduced VT-1 toxicity (Walchli *et al*, 2008), and VT-1 prolonged JNK and p38 MAPK activation of macrophage-like cells (Lee *et al*, 2007). The MAPK pathway is thus involved in proapoptotic signalling of VT-1 in stressed cell systems and the pathway is also involved in cisplatin-induced apoptosis and induced cisplatin resistance (Brozovic and Osmak, 2007). Targeting the MAPK signalling pathway could, therefore, be an additional way to reduce cisplatin-induced tumour cells resistance. We have earlier shown JNK phosphorylation in response to VT-1 treatment also in glioma cell lines (Johansson *et al*, 2006).

The treatment obstacle of acquired-cisplatin resistance in malignant plural mesothelioma and other cancers makes it necessary to find new strategies to overcome resistance. We have shown an increased expression of Gb3 in induced cisplatin-resistant MPM and NSCLC cells and a possible relation to multidrug resistance. Our results encourage the idea that Gb3-targeted therapy could be a possible approach and VT-1 either as holotoxin or by use of toxin sub-units could present a viable tool for this.
